# The clinical relevance of ultra-widefield angiography findings in patients with central retinal vein occlusion and macular oedema receiving anti-VEGF therapy

**DOI:** 10.1038/s41433-021-01553-7

**Published:** 2021-05-25

**Authors:** Luke Nicholson, Clara Vazquez-Alfageme, Piyali Sen, Namritha V. Patrao, Tunde Peto, Yit Yang, Sobha Sivaprasad, Philip G. Hykin

**Affiliations:** 1 National Institute for Health Research, Moorfields Biomedical Research Centre, London, UK; 2 The Royal Eye Unit, Kingston Hospital, Kingston, UK; 3 NetwORC UK Reading Centre, Queen’s University Belfast, Belfast, UK; 4 Wolverhampton Eye Infirmary, Wolverhampton, UK

**Keywords:** Retinal diseases, Vision disorders

## Abstract

**Aims:**

To report, using ultra-widefield angiography (UWFA) the area, distribution, and change in retinal capillary nonperfusion (RCNP) at baseline and 100 weeks in eyes with central retinal vein occlusion (CRVO) receiving anti-VEGF for macula oedema.

**Methods:**

Prospective longitudinal multi-centre cohort study. Adults with CRVO treated with anti-VEGF therapy for macular oedema underwent UWFA at baseline and week-100. The area, distribution, and change in total, peripheral and posterior pole RCNP were determined.

**Results:**

Of 153 eyes at baseline, mean area of RCNP was 34.3DA and 12 (7.8%) had ≥75DA RCNP. More than 10DA RCNP was present in the temporal periphery in 75.8% of eyes vs. 10.5% in the nasal periphery. At week-100, mean RCNP was 42.1DA with a median change from baseline of 3.3DA 95% CI [0.4, 7.3]; *p* < 0.01. Of 146 eyes with ≤10DA of posterior pole RCNP at baseline, 16/146 (11.0%) progressed to >10DA at week-100. These eyes had a median increase in total RCNP of 69.7DA [95% CI 27.2–85.4] vs 0DA [0.0–1.4]; *p* < 0.001 for those who did not, and two developed neovascular glaucoma. Larger baseline area of RCNP and history of glaucoma were risk factors for posterior pole RCNP developing.

**Conclusions:**

With UWFA, significant baseline RCNP was identified in the majority of CRVO patients, notably in the temporal periphery, but large increases over 100 weeks were uncommon. Development of >10DA posterior pole RCNP is a marker for widespread RCNP and in such cases the risk of anterior segment neovascularisation is not abolished by concomitant anti-VEGF therapy.

## Introduction

Ultra-widefield fluorescein angiography (UWFA) has increasingly replaced conventional 7 field FFA (fundus fluorescein angiography) as the optimal imaging modality in retinal vascular disease due to its ability to capture a larger retinal area and the increasing recognition of clinically significant vascular irregularities in the retinal periphery. However, despite such promise, few prospective studies have captured UWFA images in large cohorts of patients with retinovascular disease, particularly central retinal vein occlusion (CRVO), partly due to the technical demands of image acquisition, potential risks of intravenous fluorescein injection, and cost.

Conventional seven field imaging identified that patients with non-ischaemic CRVO typically present with less than 10 disc areas (DA) of retinal nonperfusion and the presence of more than 10DA suggests an increased risk of neovascularisation with 34% of eyes developing an ischaemic phenotype within 3 years [1]. However, with UWFA we recently reported that untreated eyes with <10DA RCNP did not develop neovascular complications but those with >30DA had >20% risk, increasing to 80% risk with >75DA [1].

Anti-vascular endothelial growth factor (VEGF) is the mainstay of treatment for patients with macular oedema secondary to CRVO, however, the incidental effect of this treatment on the natural history of retinal nonperfusion is unclear, largely because the treatment regimen, initial loading frequency, and drug type are determined by the CMO, investigator preference and cost rather than retinal perfusion. Nevertheless, it is an important question as most patients are treated with anti-VEGF therapy for the duration of their macular oedema and it may significantly modify clinical and imaging findings.

CRUISE reported 3.9% of eyes treated with repeated ranibizumab therapy for macula oedema secondary to non-ischaemic CRVO developed anterior segment neovascularisation by 12 months but did not report progression of retinal nonperfusion on UWFA [2]. However, in patients with ischaemic CRVO, the prevalence of neovascularisation may be significantly masked during anti-VEGF therapy and the risk of neovascularisation is not permanently ameliorated with anti-VEGF treatment [3]. The clinical diagnosis of conversion to an ischaemic CRVO was reported in 5.4% of LEAVO study patients [4].

However, the prevalence of angiographic evidence of progressive RCNP was believed to be higher as on-going anti-VEGF treatment may have masked clinical manifestations e.g. retinal haemorrhages and cotton wool spots in some cases, leading to so called ‘silent’ progression. Furthermore, in an earlier CRVO study using UWFA, we showed that 84.6% of treatment naive eyes with >10DA of RCNP in the posterior pole, defined as an area of 10 disc diameters centred on the fovea, secondary to CRVO experienced neovascular complications [1]. Therefore, progression of RCNP to involve more than 10DA of the posterior pole likely represents an important finding that may merit early intervention.

The objectives of this study were (i) to report the area and distribution of RCNP at baseline and 100 weeks using UWFA in a large prospective patient cohort, (ii) to report progression of peripheral and posterior pole (>10 DA) RCNP while on concomitant anti-VEGF treatment for macular oedema, (iii) identify associated risk factors for conversion and (iv) suggest clinically useful management guidance.

## Methods

This is a prospective, longitudinal, multi-centre, image analysis, cohort study of eyes imaged with UWFA within the LEAVO trial, a multicentre phase 3 double-masked randomised controlled non-inferiority trial comparing the clinical effectiveness of intravitreal therapy with ranibizumab (Lucentis) vs aflibercept (Eylea) vs bevacizumab (Avastin) for macular oedema secondary to CRVO over a 100 week period conducted between 2014 and 2018. This study was approved by the United Kingdom National Ethics Committee Service (14/LO/1043) and the study was conducted in accordance with the tenets of the Declaration of Helsinki. Written informed consent was obtained from participants prior to entry into the study.

### Participants

Adults with macular oedema secondary to CRVO of less than 12 months duration with best corrected visual acuity Early Treatment Diabetic Retinopathy Study letter score in the study eye between 19 and 78 and spectral domain optical coherence tomography central subfield thickness ≥320 μm or equivalent were included in the LEAVO study. In this sub-study, 235 participants with UWFA were included. Participants received a loading dose of four mandated monthly anti-VEGF injections followed by 4–8 weekly assessments and retreatment criteria based on visual acuity and OCT central subfield thickening.

### Eligibility criteria

The eligibility criteria for the LEAVO study have been described [4]. For this prospective UWFA sub-study eligibility, only participants who had UWFA performed using the Optos ultra-widefield system (Optos Plc, Dumfermline, Scotland) with gradeable angiograms at baseline and 100 weeks were included. Each fovea centred angiogram image was assessed and only included if images were sharp enough to distinguish areas of perfused from nonperfused retina and were corrected for projection artefact.

### Image acquisition

The UWFA images were obtained using the Optos ultra-widefield system observing a standard protocol. All images were captured by specialist photographers who had been trained and certified by Optos representatives. A single investigator (LN) identified the best macula centred FA image in the arteriovenous phase from the series of each eligible eye. The area imaged in the baseline and week-100 images was similar.

### Image processing

The concentric rings template was applied to each image [5]. This validated method incorporates a macular ring and five additional concentric rings (rings 1–5), each with a 2.5 disc diameters (DD) increment in radius. The concentric rings were placed at the centre of the fovea and the margin of ring M was at the centre of the optic disc. The distance between the centre of the disc and the centre of the fovea was the same as the radius of the first ring, which was 2.5DD so that a standardised measure was obtained. Each of the six rings (Ring M and 1–5) are divided into 12 segments. Each segment is graded as ungradable, nonperfused or perfused if 50% or more of the segment is involved [5]. If retinal haemorrhages prevent grading of ≥50% of a segment, the entire segment is deemed ungradable The area of each segment in each ring was corrected based on the enlargement factor identified using 3-D printed model eyes [6]. The area per segment was 1.85DA for Ring M, 5.21DA for Ring 1, 7.78DA for Ring 2, 9.47DA for Ring 3 and 10.36DA for Ring 4 [1].

Images used were fovea-centred and montages of steered images were not used as the distortion with steered images is unclear. Based on our validation study, ring 5 was largely ungradable. The superior and inferior segments of rings 3 and 4 were typically ungradable due to the nature of the ultra-wide field image having better clarity in the horizontal meridian [5].

### Image analysis

**Fig. 1:**
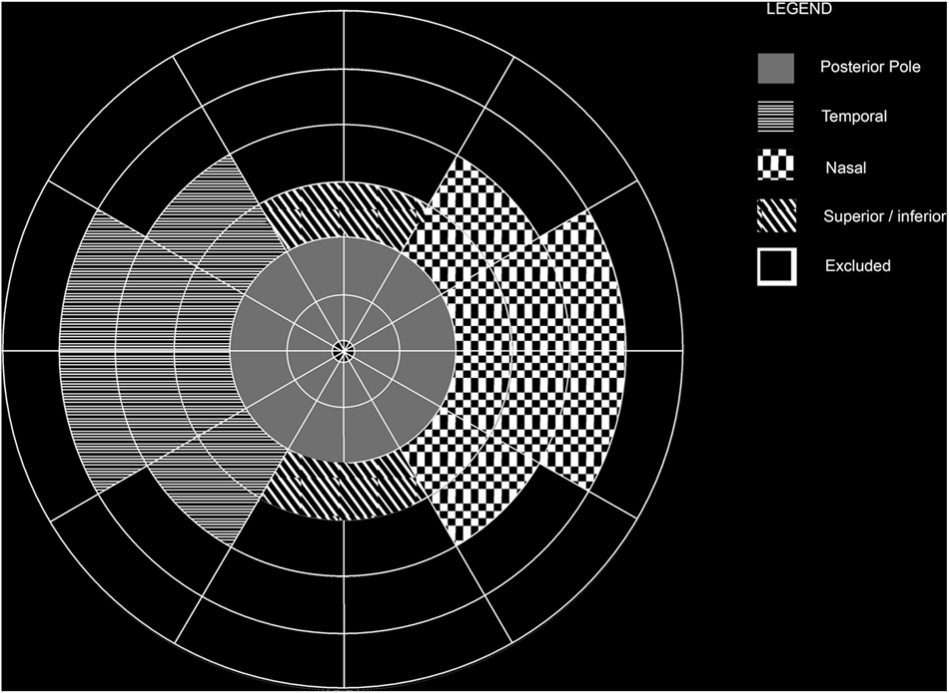
The modified concentric rings template with the boundaries within the modified concentric rings for the posterior pole, temporal periphery, nasal periphery, superior and inferior periphery in this study in a right eye. For a left eye, the nasal and temporal areas are flipped.

Images were graded by two consultant medical retinal specialists (LN and CVA) and average values used. Baseline and week-100 images were graded separately. Data were obtained for total area of retinal nonperfusion, posterior pole, temporal periphery and nasal peripheral nonperfusion, the location of which is described in Fig. 1. The number of eyes with >10DA of posterior pole nonperfusion at baseline and week-100 were also determined because this degree of nonperfusion has been previously identified to be closely related to neovascularisation [1].

### Data collection

Data were collected for age, sex, duration of CRVO, visual acuity, central subfield thickness, number of intravitreal anti-VEGF injections, longest interval between injections, anterior segment neovascularisation and neovascular glaucoma. Data were also collected for history of glaucoma, cardiovascular disease, haematological disease, and neoplasia at baseline.

### Statistical analysis

Intraclass correlation coefficient for average measurements was used to describe the intergrader agreement. Comparisons between the baseline versus week-100 results and temporal versus nasal area of nonperfusion were analysed using the Wilcoxon rank sum test and the change in retinal nonperfusion expressed as the Hodges–Lehmann median difference. Backward stepwise binary logistic regression was used to study variables for progression to >10DA of posterior pole nonperfusion with eyes that did not. Statistical significance was set as 0.05.

## Results

From 235 participants with UWFA,184 eyes of 184 participants had both baseline and week-100 UWFA. Thirty-one eyes were excluded due to poor quality and ungradable images at baseline or week-100 resulting in a total of 153 eyes and 306 images (baseline and week 100) included in the final analysis. The median age was 69 years (interquartile range {IQR} 18), 83 (54.2%) were female, and 86.3% had a duration of CRVO of less than 3 months prior to entry into the study. The median baseline visual acuity was 57 letters (IQR, 19.3) and OCT central subfield thickness was 691 µm (IQR, 392). Thirteen eyes (8.5%) had a history of glaucoma at baseline.

The intraclass correlation coefficient between graders for 306 images was 0.982, representing an almost perfect agreement [7]. The mean baseline total area of RCNP was 34.3DA 95% CI [28.8, 39.8]. At baseline, 36 eyes (23.5%) had <10 DA of RCNP, 45 (29.4%) between 10 and 30DAs of RCNP, 60 (39.2%) between >30 and 75DA while 12 (7.8%) had more than 75DA of RCNP on UWFA.

**Fig. 2:**
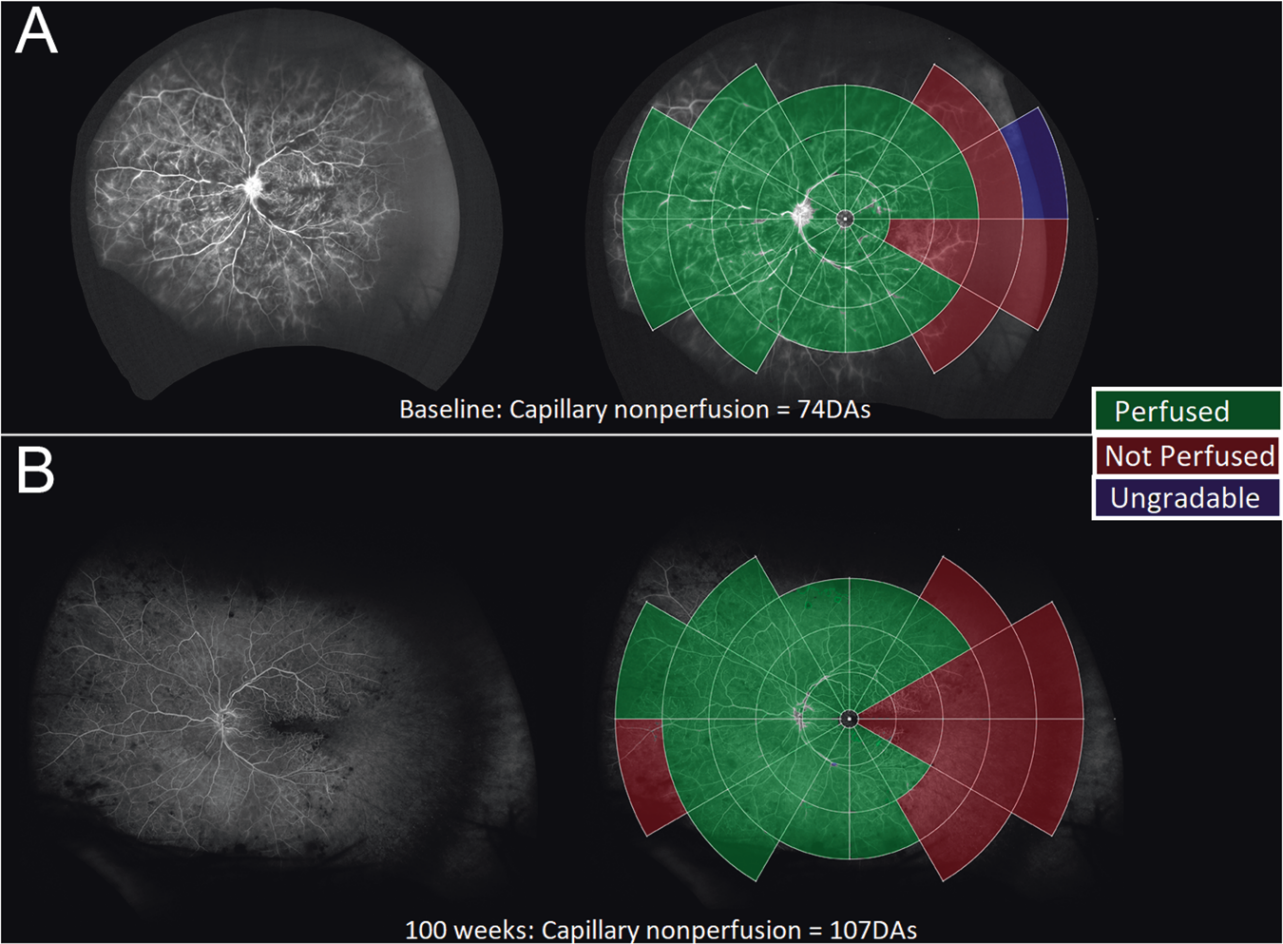
An example of a patient with central vein occlusion with the modified concentric rings template overlaid at baseline and the 100-week visit manifesting significant temporal retinal capillary nonperfusion. The top image (**A**) represents the baseline ultra-widefield angiogram and on the right, with the overlaid template demarcating the areas of perfusion and nonperfusion which is more evident temporally. The bottom image (**B**) represents the ultra-widefield angiogram at 100 weeks and on the right, with the overlaid template showing marked increase in nonperfusion, mainly in the temporal retina.

116 (75.8%) eyes had >10DA of temporal peripheral RCNP with only 16 (10.5%) eyes having >10DA of nasal peripheral RCNP. The median difference between the temporal and nasal peripheral RCNP was 25.0DA 95% CI [22.2, 28.9]; *p* < 0.001. It was also noted that eyes with temporal RCNP commonly manifest a triangular segment of RCNP pointed towards the fovea. (Fig. 2).

The mean total area of RCNP at week-100 was 42.1DA 95% CI [35.5, 48.8] with a median change compared to baseline of 3.3DA 95% CI [0.4, 7.3]; *p* = 0.008. At 100 weeks, 26 eyes (17.0%) had <10 DA of RCNP, 44 (28.8%) between 10 and 30DA, 61 (39.9%) between >30 and 75DA and 22 (14.4%) had more than 75DA of RCNP on UWFA. Table 1 details the quantity and change in RCNP within each area.

**Table 1:** The number of eyes with greater than 10-disc areas (DA) of nonperfusion and the associated mean and 95% confidence interval of the area of nonperfusion within the total area, posterior pole, temporal periphery, nasal periphery, superior periphery and inferior periphery at baseline and week-100.

	Number of eyes with >10DA of regional nonperfusion at baseline, *n* (%)	Mean area of nonperfusion, DA, at baseline	Number of eyes with >10DA of regional nonperfusion at Week-100, *n* (%)	Mean area of nonperfusion, DA, at week-100	Median difference between area of nonperfusion at baseline and week-100
Total	118 (77.1)	34.3 [28.8, 39.8]	128 (83.7)	42.1 [35.5, 48.8]	3.3 [0.4, 7.3]; *p* = 0.008
Posterior pole	7 (4.6)	1.3 [0.4, 2.1]	21 (13.6)	2.9 [1.7, 4.1]	0.0 [0.0, 0.0]; *p* < 0.001
Temporal periphery	116 (75.8)	28.9 [25.4, 32.4]	127 (83.0)	33.0 [29.1, 37.0]	2.8 [0.2, 5.6]; *p* = 0.008
Nasal periphery	16 (10.5)	3.1 [1.3, 4.9]	24 (36.7)	4.8 [2.7, 6.9]	0.0 [0.0, 0.0]; *p* = 0.02
Superior periphery^a^	3 (2.0)	0.51 [0.12, 0.90]	4 (2.6)	0.61 [0.19, 1.0]	0.0 [0.0, 0.0]; *p* = 0.50
Inferior periphery^a^	4 (2.6)	0.53 [0.13, 0.94]	4 (2.6)	0.76 [0.31, 1.2]	0.0 [0.0, 0.0]; *p* = 0.14

^a^The superior and inferior regions are likely to report reduced values given the limited field of view imaged.

Of 146 eyes with ≤10DA of posterior pole RCNP at baseline, 16 (11%) progressed to involve more than 10DA by week-100. The baseline characteristics of eyes that showed such progression and those that did not are described in Table 2. Using binary logistic regression for the conversion to >10DA of posterior pole RCNP by week-100, only a larger baseline total area of RCNP (Odds Ratio {OR} 1.02 per DA 95% CI [1.00–1.04]; *p* = 0.04) and history of glaucoma at baseline (OR 24.82 95% CI [4.16–147.99]; *p* < 0.001, were found to be significant. Other variables studied, age, sex, duration of CRVO, baseline visual acuity, central subfield thickness, total number of anti-VEGF treatment during the study, longest interval between anti-VEGF treatment during the study, history of cardiovascular disease, history of haematological disease and history of neoplasia at baseline, were all not found to be statistically significant.

**Table 2:** Demographics characteristics for eyes with progressed to >10DA of posterior pole nonperfusion and eyes that remained <10DA of posterior pole nonperfusion over 100 weeks from eyes with <10DA of posterior nonperfusion at baseline, *n* = 146.

Characteristics	Conversion to > 10DA of posterior pole nonperfusion at week-100, *n* = 16	Remained < 10DA of posterior pole nonperfusion at week-100, *n* = 130
Age, years^a^	74 [68.6, 84.4]	68 [65, 71]*
Female^b^	8 (50%)	69 (53%)
Baseline visual acuity, letters^a^	53 [44.1, 59.0]	58 ([56.3, 60.7]
CRVO < 3 months duration^b^	13 (81.3%)	113 (86.9%)
Baseline total area of nonperfusion, DA^a^	50.5 [31.5, 69.6]	27.8 [23.6, 32.0]*
Change in RNP, DA^a^	69.7 [21.6, 85.4]	0 [0, 0.4]*
Number of anti-VEGF injections	14 [10.0, 14.4]	11.5 ([10.0, 13.0]
Longest interval between injections, days	96 [79.6, 142.9]	84 [78.8, 94.1]
Glaucoma at baseline^b^	6 (37.5%)	5 (3.8%)*

^a^Data presented as mean and 95% confidence interval of the mean.

^b^Data presented as number (percentage).

*P < 0.05.

**Fig. 3:**
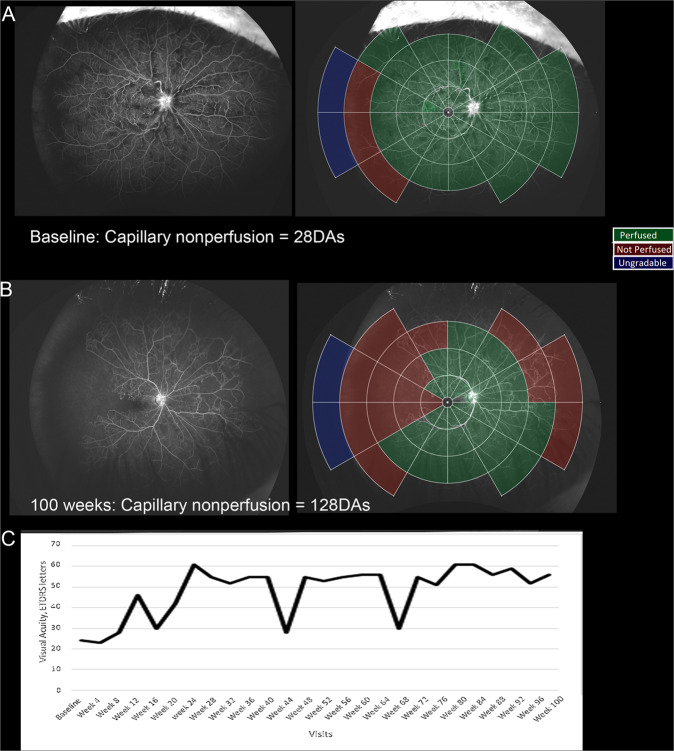
An example of a participant with significant increase in retinal nonperfusion with no persistent reduction in visual acuity gains. The top image (**A**) represents the baseline ultra-widefield angiogram and on the right, with the overlaid template demarcating the areas of perfusion and nonperfusion. The middle image (**B**) represents the ultra-widefield angiogram at 100 weeks and on the right, with the overlaid template. The bottom image (**C**) displays the participant’s individual visual acuity letter score over the 100 weeks while on intravitreal anti-VEGF therapy for macular oedema.

Of the 16 eyes that progressed to ≥10DA of posterior pole RCNP at 100 weeks, there was a median 69.7 DA increase in total RCNP compared to zero DA increase in those that did not and two, developed anterior segment neovascularisation. (Fisher exact test: *p* = 0.01). Eight of these 16 eyes (50%) had persistent ≥10 letter loss in visual acuity from their best recorded study visual acuity, compared to 25/130 (19.2%) who did not develop ≥10DA posterior pole RCNP (Fishers exact test: *p* = 0.01). Eight eyes of the 16 (50%) that progressed to >10DA of posterior pole RCNP at 100 weeks did not manifest neovascular complications or permanent visual acuity change. This is illustrated by Fig. 3 where there is a significant increase in total RCNP, from baseline 28DA to week 100, 128DA, including more than 10DA posterior pole increase but no permanent change in visual acuity occurred whilst receiving pro re nata intravitreal anti-VEGF treatment for macular oedema.

## Discussion

This is the first prospective study to perform UWFA at baseline and 100 weeks, in a large cohort of eyes with CRVO receiving anti-VEGF for macular oedema. The overall quantity of RCNP identified on UWFA was greater at baseline (34DA) than with conventional 7 field FFA [8], and likely consistent with a population eligible for anti-VEGF therapy for associated macula oedema with no or only a minor increase in RCNP in most eyes through follow-up (median 3.3.DAs), possibly due to concomitant anti-VEGF therapy. Such change was global but mainly driven by the temporal periphery (see Fig. 1).

We describe a novel finding that retinal nonperfusion in CRVO predominantly affects the temporal compared to the nasal retina, a feature that persisted through 100 weeks. It is acknowledged that the Optos system has better coverage of the horizontal meridian as opposed to the vertical, and therefore, superior and inferior retinal assessment were limited despite the use of Optos trained and certified imaging technicians. Additionally, steered images were not used, due to technical issues with image alignment during the study, but nevertheless nasal quadrant RCNP was markedly less than temporal retina despite both quadrants being imaged equally. We postulate the reason behind this relates to the temporal part of the horizontal raphe watershed zone being most susceptible to nonperfusion and the distance from the optic disc. As CRVO affects the flow within the retina, the fact that the temporal retina is furthest from the optic disc also may be a contributory reason [9–11].

Interestingly, a small number of eyes 7.8% (12/153) had larger areas of RCNP (>75DA) identified with baseline UWFA likely consistent with the broad visual acuity study eligibility criteria that purposefully permitted recruitment of eyes with significant RCNP to ensure the study was representative of the population at large.

Of note the number of eyes affected had increased to 14.4% (22/153) at week 100, despite the use of anti-VEGF therapy with 4–8 weekly follow up and sufficient treatment regularity to maintain initial visual acuity gains through 100 weeks, supporting the previously reported finding that anti-VEGF may delay or modify but does not abolish the risk of progressive retinal ischaemia and the risk of anterior segment or retinal neovascularisation. However direct comparison of this study cohort with the population in the Central Vein Occlusion Study in which 34% developed an ischaemic CRVO at 3 years requires caution due to the heterogeneous clinical presentation of CRVO, differing study durations and exclusion criteria. Additionally, the risk factors for CRVO may be better controlled today than two decades ago and participants in our cohort were diagnosed and managed at an earlier stage in the disease. This is substantiated by the fact that more recent CRVO series looking at conversion to ischaemia without the influence of anti-VEGF reported 12.5 and 15% conversion rates [12–14].

Furthermore, such cases overlapped significantly with the group of patients who developed >10DA of posterior pole RCNP by 100 weeks. In fact, 14 of the 16 with the latter had greater than 75DA nonperfusion at 100 weeks. Thus >10DA posterior pole nonperfusion may be a useful indicator of peripheral nonperfusion. For instance, if present in a patient in whom the peripheral retina could not be visualised due to e.g. vitreous haemorrhage or poor pupil dilation or simply was visible but not readily assessable due to the presence of multiple retinal haemorrhages, it would provide strong evidence that significant peripheral RCNP was indeed present and help in deciding whether additional anti-VEGF therapy or panretinal photocoagulation was indicated.

Additionally, 50% of cases that progressed to involve >10DA nonperfusion did not manifest neovascularisation or a permanent reduction in vision. This is concerning as while on intravitreal anti-VEGF treatment, a ‘silent’ progression of ischaemia can occur in eyes without change in visual acuity and remain undetected unless angiography is performed. Given the incidence of neovascularisation following cessation of anti-VEGF therapy in ischaemic CRVO in the RAVE study, ‘silent’ progression to an ischaemic retina stresses the importance of angiography if anti-VEGF is ceased or temporarily deferred. This finding is also consistent with the reports from the RAVE study, reporting sustained improvement in visual acuity when in the pro re nata anti-VEGF injection phase and in a subsequent report describing progressive increase in nonperfusion in the same cohort [3, 15]. Comparing eyes that remained stable and eyes that progressed to >10DA of posterior nonperfusion, see Table 2, eyes that progressed to involve >10DA of posterior nonperfusion received more intravitreal anti-VEGF treatments, 14 as opposed to 11.5 in eyes that remain stable and there was no difference in the longest interval between injections, again not suggestive of anti-VEGF therapy significantly preventing conversion to an ischaemic CRVO.

We have identified larger baseline area of retinal nonperfusion and more significantly, a history of glaucoma at baseline to have greater odds of progression to posterior pole nonperfusion (>10DA) which reflects previous findings of glaucoma conferring a risk factor for the incidence of CRVO [16, 17]. The mechanism may be related to optic nerve changes such as a larger cup to disc ratio which has been found to be related to CRVO or a common microvascular aetiology [18].

We acknowledge the limitations that three different anti-VEGF agents were used and that the retreatment criteria were based on visual acuity and macular oedema rather than change in retinal nonperfusion. Comparing the change in retinal nonperfusion after 100 weeks between the three treatment arms did not reveal a statistically significant difference, *p* = 0.087, however, randomisation did not stratify for baseline retinal nonperfusion and the study was not designed to evaluate this primarily, therefore, strong conclusions on this cannot be made.

In conclusion, with UWFA, more baseline (mean 34.3) and week 100 (mean 42.1) RCNP, notably in the temporal quadrant was identified compared to conventional FA imaging. During two years of follow-up, eleven percent of eyes experienced progression of posterior pole RCNP (>10DA) and anterior segment neovascularisation occurred in 2 despite intravitreal anti-VEGF for macular oedema. More than 10 DA posterior pole RCNP is a useful marker of significant widespread RCNP and may inform ongoing management decisions. Furthermore, half of such cases did not manifest a permanent reduction in vision. Therefore, in addition to clinical examination to exclude early signs of neovascularisation, angiographic detection of posterior pole RCNP, especially if the peripheral retina cannot be visualised or UWFA is unavailable, may prompt additional treatment to prevent neovascularisation especially in cases where anti-VEGF therapy has been interrupted or stopped.

## Summary

### What was known before

- 34% of eyes with central retinal vein occlusion develop an ischaemic phenotype in 3 years.

- More than 10DA of posterior pole nonperfusion is associated with a higher incidence of neovascularisation.

- Anti-VEGF does not ameliorate the development of neovascularisation.

### What this study adds

- The average total area of retinal nonperfusion on ultra-widefield imaging is 34.3DA.
- The temporal periphery commonly manifests retinal nonperfusion in eyes with CRVO.
- 11% of eyes progress to involve more than 10DA of posterior pole nonperfusion while on anti-VEGF therapy for macular oedema.
